# Microbial production of non‐canonical amino acids: An annotated selection of World Wide Web sites relevant to the topics in microbial biotechnology

**DOI:** 10.1111/1751-7915.14376

**Published:** 2023-11-29

**Authors:** Lawrence P. Wackett

**Affiliations:** ^1^ McKnight Professor, Department of Biochemistry, Molecular Biology & Biophysics BioTechnology Institute, University of Minnesota St. Paul Minnesota USA

Non‐canonical amino acids in anti‐microbial peptides

https://www.ncbi.nlm.nih.gov/pmc/articles/PMC9746297/

This report deals with the incorporation of non‐canonical amino acids into antimicrobial peptides, which can extend the lifetime of the peptides by making them relatively recalcitrant to enzymatic degradation.

Non‐canonical amino acids review

https://www.ncbi.nlm.nih.gov/pmc/articles/PMC10534643/

This paper reviews research related to non‐canonical amino acids and methods for the expansion of the genetic code.

Tyrosine sulfation

https://www.nature.com/articles/s41467‐022‐33111‐4

In this report, cells were engineered to carry out site‐specific insertion of sulfated tyrosine residues in proteins.

Addicting bacteria to non‐canonical amino acids

https://www.aiche.org/sbe/conferences/synthetic‐biology‐engineering‐evolution‐design‐seed/2015/proceeding/paper/single‐plasmid‐system‐addict‐bacteria‐non‐canonical‐amino‐acids

This study showed it was possible to evolve enzymes with a high dependence on non‐canonical amino acid incorporation.

Scalable production of non‐canonical amino acids

https://www.sbir.gov/node/2214999

There are various applications in which the introduction of non‐canonical amino acids would enhance the usefulness of certain peptides and proteins. The applications require scale‐up and this page describes an effort to fund the development of industrial‐scale production.

Non‐canonical amino acids for protein stabilization

https://academic.oup.com/peds/article/doi/10.1093/protein/gzad003/7075689

Many industrial enzymes are thermostable which typically imparts stability under many working conditions. This paper discusses how the insertion of non‐canonical amino acids can increase thermostability.

Photocatalysis for non‐canonical amino acids

https://phys.org/news/2023‐07‐scientists‐unveil‐synergistic‐method‐non‐canonical.html

This article is a commentary on research designed to use photocatalysis to create non‐canonical amino acids stereoselectively.

Enzyme engineering with non‐natural amino acids

https://portlandpress.com/bioscirep/article/42/8/BSR20220168/231590/Engineering‐of‐enzymes‐using‐non‐natural‐amino

This recent review paper describes the engineering of proteins containing non‐canonical amino acids.

Amino acids shaping the genetic code

https://www.news‐medical.net/news/20230227/New‐insights‐into‐how‐amino‐acids‐shaped‐the‐genetic‐code‐of‐ancient‐microorganisms.aspx

This study posits that early amino acids that formed before cellular life were selected for the ability, when oligomerized, to fold into certain shapes. Many of the early, pre‐life, amino acids were non‐canonical.

Commercial non‐natural amino acids

https://www.tocris.com/product‐type/unnatural‐amino‐acids

This commercial site provides a variety of non‐canonical amino acids for purchase, facilitating research.

Genetic code expansion products

https://www.addgene.org/collections/genetic‐code‐expansion/

This commercial site provides a variety of non‐canonical amino acids for purchase, facilitating research.

Transaminases making non‐natural amino acids

https://chemrxiv.org/engage/chemrxiv/article‐details/64de820d694bf1540c84d805

Non‐canonical amino acids can be made enzymatically using transaminases. This study used the enzymes to construct azacyclic amino acids.

